# *Trypanosoma cruzi trans*-Sialidase in Complex with a Neutralizing Antibody: Structure/Function Studies towards the Rational Design of Inhibitors

**DOI:** 10.1371/journal.ppat.1002474

**Published:** 2012-01-05

**Authors:** Alejandro Buschiazzo, Romina Muiá, Nicole Larrieux, Tamara Pitcovsky, Juan Mucci, Oscar Campetella

**Affiliations:** 1 Institut Pasteur de Montevideo, Unit of Protein Crystallography, Montevideo, Uruguay; 2 Institut Pasteur, Department of Structural Biology and Chemistry, Paris, France; 3 Instituto de Investigaciones Biotecnológicas, Universidad Nacional de San Martín, San Martín, Buenos Aires, Argentina; 4 Consejo Nacional de Investigaciones Científicas y Técnicas, Buenos Aires, Argentina; University of Wisconsin-Madison, United States of America

## Abstract

*Trans*-sialidase (TS), a virulence factor from *Trypanosoma cruzi*, is an enzyme playing key roles in the biology of this protozoan parasite. Absent from the mammalian host, it constitutes a potential target for the development of novel chemotherapeutic drugs, an urgent need to combat Chagas' disease. TS is involved in host cell invasion and parasite survival in the bloodstream. However, TS is also actively shed by the parasite to the bloodstream, inducing systemic effects readily detected during the acute phase of the disease, in particular, hematological alterations and triggering of immune cells apoptosis, until specific neutralizing antibodies are elicited. These antibodies constitute the only known submicromolar inhibitor of TS's catalytic activity. We now report the identification and detailed characterization of a neutralizing mouse monoclonal antibody (mAb 13G9), recognizing *T. cruzi* TS with high specificity and subnanomolar affinity. This mAb displays undetectable association with the *T. cruzi* superfamily of TS-like proteins or yet with the TS-related enzymes from *Trypanosoma brucei* or *Trypanosoma rangeli*. In immunofluorescence assays, mAb 13G9 labeled 100% of the parasites from the infective trypomastigote stage. This mAb also reduces parasite invasion of cultured cells and strongly inhibits parasite surface sialylation. The crystal structure of the mAb 13G9 antigen-binding fragment in complex with the globular region of *T. cruzi* TS was determined, revealing detailed molecular insights of the inhibition mechanism. Not occluding the enzyme's catalytic site, the antibody performs a subtle action by inhibiting the movement of an assisting tyrosine (Y_119_), whose mobility is known to play a key role in the *trans*-glycosidase mechanism. As an example of enzymatic inhibition involving non-catalytic residues that occupy sites distal from the substrate-binding pocket, this first near atomic characterization of a high affinity inhibitory molecule for TS provides a rational framework for novel strategies in the design of chemotherapeutic compounds.

## Author Summary

Chagas' disease, or American trypanosomiasis, is an endemic illness that affects approximately 8 million people in Latin America. The etiologic agent is the protozoan parasite *Trypanosoma cruzi*. To survive in the mammalian host and invade its cells, leading to the chronic infection, the parasite incorporates a charged carbohydrate (sialic acid). However, the parasite is unable to synthesize sialic acid, having to scavenge it from the host's sialo-glycoconjugates, through a transglycosylation reaction catalyzed by the enzyme *trans*-sialidase, which is unique to these organisms. We have obtained a monoclonal antibody that fully inhibits *T. cruzi trans*-sialidase actually being, at the best of our knowledge, the most potent inhibitor available. We now report a complete characterization of this neutralizing monoclonal antibody, at the functional and molecular levels. The antibody displays very high affinity and specificity for the *T. cruzi* enzyme, labels the parasites' surface and effectively blocks its sialylation and host cell invasion capacities. The determination of the 3D structure of the enzyme-antibody immunocomplex by X ray diffraction, allowed us to unveil the inhibition mechanism, providing clues for rational drug design. Given that sialidases are virulence factors in several pathogenic microorganisms, the reported data shall help to expand informative knowledge in this area.

## Introduction

Chagas' disease, the American trypanosomiasis, is a chronic disabling parasitic disease caused by the flagellate protozoon *Trypanosoma cruzi.* With an estimated global burden of 100 million people at risk, 8 million already infected, and approximately 40,000 new cases/year, Chagas' disease represents a major health and economic problem in Latin America [1]. The infection is naturally transmitted by triatomine vectors (“kissing bugs”), from the south of the USA to the southern region of South America, although chagasic patients are in fact dispersed worldwide due to migrations. Patients can also transmit the disease either by *in utero* infection leading to the congenitally acquired disease or by accidental transmission through contaminated blood. The acute infection is characterized by patent parasite burden. During this initial stage, *T. cruzi* induces several alterations in the infected mammal including intense polyclonal activation of lymphocytes [2], transient thymic aplasia [3], [4] and other clinical hematological findings [5], [6]. The majority of the patients control the parasitemia, survive the acute phase, and enter into an indeterminate form of the disease that may last for many years or even indefinitely [1]. Up to 20 years after the infection, ∼35% of patients develop different pathologies, such as cardiomyopathy, peripheral nervous system damage, and/or dysfunction of the digestive tract [1].

Sialic acids have proven to be crucial during the parasite's life cycle and survival in the mammalian host [7]–[10]. However, *T. cruzi* is unable to perform *de novo* synthesis of sialic acids [11]. This family of nine-carbon carbohydrates, is actually scavenged from the host's glycoconjugates, through a glycosyl-transfer reaction mediated by *trans*-sialidase (TS), a modified sialidase expressed by the parasite. In this way, the surface of the parasite becomes rapidly sialylated, with mucins being the main sialyl acceptors, in a process that allows the parasite to evade its destruction by serum factors [9], [10]. TS activity is also involved in host cell attachment and invasion [7], [8], as well as in parasite escape from the parasitophorous vacuole into the cytoplasm, where the parasite replicates [12].

In the trypomastigote stage, TS is a glycosylphosphatidylinositol-anchored non-integral membrane protein [13], actively released to the extracellular milieu, leading to a systemic distribution of the enzyme through the bloodstream. Its half-life in blood is significantly extended due to the presence of a *C*-terminal repetitive domain named SAPA [14]. TS activity is detectable in the bloodstream of infected humans and mice, until antibodies able to neutralize its catalytic activity are elicited [15]. The systemic distribution of TS is associated with several pathologies observed during the early steps of infection including depletion of thymocytes [16], absence of germinal centers in secondary organs [17] and thrombocytopenia and erythropenia [5], [6], all alterations that can be prevented by the passive transfer of TS-neutralizing antibodies [17], [18]. In fact, administration of the enzyme in mice before *T. cruzi* challenge, leads to more severe evolution of the infection [19]. These finding are also consistent with the fact that increased shedding of the enzyme correlates with increased virulence of the corresponding parasite strains [20].

TS has thus been identified as a potential target for drug discovery and design. Added to its key roles in host response evasion, cell invasion and pathogenesis, TS is not present in the mammalian host. The development of suitable drugs to treat/prevent Chagas' disease is urgently needed [21]. Only two compounds, benznidazol and nifurtimox, are currently available for treating both acute and chronic infections. These drugs are far from being optimal: fairly toxic, they trigger serious side effects, while also showing suboptimal efficacy in a high proportion of patients. The emergence of resistant parasite strains adds a concerning issue [22]. Several attempts to obtain suitable TS inhibitors have been made, especially once its 3D structure became available [23], [24]. However, only low affinity molecules have been obtained so far [25], [26], some of them toxic in *in vivo* assays [27], ultimately suggesting that further and more active efforts must be pursued.

We have obtained a TS-neutralizing mouse monoclonal antibody (mAb 13G9) that displays very high affinity and specificity towards the *T. cruzi* enzyme. This mAb is able to prevent immune system and hematological abnormalities, even when assaying highly virulent parasites under lethal infection conditions [5], [17]. We now report an extensive functional characterization of mAb 13G9, as well as the crystal structure of the 13G9-TS binary complex. The molecular features of the inhibitory mechanism are unveiled, providing novel insight for the development of TS inhibitors, which might also be relevant for related neuraminidases in other pathogens.

## Results

### Biochemical Characterization of the TS-neutralizing Monoclonal Antibody

**Figure 1:**
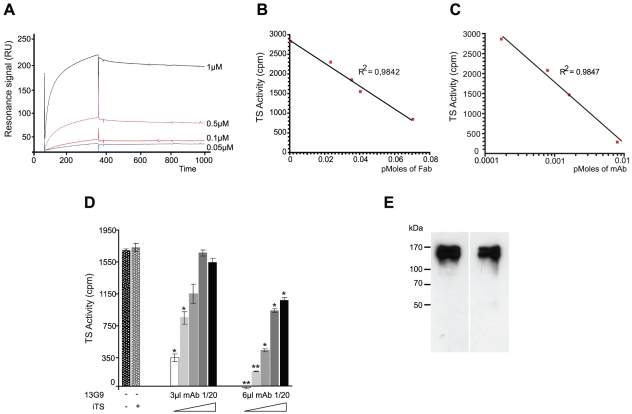
Biochemical characterization of the TS-13G9 mAb interaction. **A**) Surface plasmon resonance analysis of TS-mAb 13G9 interaction kinetics. mAb 13G9 was immobilized onto a CM5 sensor chip and the indicated concentrations of TS were injected in the mobile phase. **B, C**) mAb 13G9 inhibition of TS activity. TS (2 ng) was mixed with increasing amounts of purified Fab (**B**), or whole 13G9 IgG (**C**) and remnant TS activity was assayed. **D**) Competition assay for mAb 13G9-TS binding. TS activity was assayed (2 ng, 30 min) in the presence of the neutralizing mAb (3 and 6 µl of hybridoma culture supernatant diluted 1/20) and increasing amounts (from 0 to 8 ng) of the inactive TS (iTS) were added. Student's *t* test was used. * *p*<0.05; ** *p*<0.005 comparing against TS activity without mAb addition **E**) Specificity of the mAb 13G9. Trypomastigotes were biotinylated, washed and mAb 13G9 added to pull-down reacting proteins. Western blots were developed with anti-SAPA (left) and streptavidin (right).

Mice were immunized with a TS recombinant protein (Δ1443TS), identical to the wt except it includes a deletion of a non-neutralizing epitope. Δ1443TS retains full enzymatic activity, while avoiding the otherwise typical delay in eliciting TS-neutralizing antibodies [28], [29]. Hybridomas were screened by TS-inhibition assay [30] and the 13G9 clone secreting a TS-neutralizing mAb (IgG_2aκ_) was obtained. The specificity of this mAb was confirmed by the absence of reactivity against the closely related sialidase from *Trypanosoma rangeli* and the TS from *Trypanosoma brucei* (data not shown). As depicted in Figure 1A, this mAb showed high affinity for the *T. cruzi* TS (K_D_ ∼7.2×10^−10^ M) as calculated from the kinetic constants determined by surface plasmon resonance. In agreement, isothermal titration calorimetry assays indicated an equilibrium dissociation constant lower than 10^−9^ M (raw data not shown).

The mAb was purified by Protein A-affinity chromatography from filtered hybridoma supernatants. This purified material was further subjected to anionic chromatography (Figure S1). The mAb eluted as a single peak as evaluated both by TS-neutralizing activity (not shown) as well as by TS recognition in dot-blot assays (Figure S1). The same sequence was found in several mRNAs encoding for the antibody (not shown), in support of a clonal nature of the hybridoma. Purified mAb was proteolized with papain to generate the Fab fragment. Inhibitory activity of the fragment was determined and compared with that from the whole IgG protein (Figure 1, panels B and C). Although the full-length mAb appears to have a higher inhibitory activity (half maximal inhibitory concentration IC_50_ 5.6×10^−11^ M), its Fab fragment still retains a nanomolar IC_50_ (1.6×10^−9^ M), clearly conserving its antigen-binding mechanism. These high inhibitory potencies are consistent with the apparent dissociation constant determined by surface plasmon resonance (see above), even though IC_50_ figures cannot be compared with affinity constants in absolute terms at this point (allosteric effects, or yet mixed inhibition mechanisms, may flaw a linear relationship). The purified Fab proved to be fairly unstable when non-complexed to TS, requiring immediate use for biochemical characterizations. This may be one of the main reasons for the observed inhibitory potency decrease compared to the entire immunoglobulin molecule. The Fab's instability precluded its use for further *in vivo* and *in vitro* biologic assays.

*T. cruzi* TS belongs in fact to a huge superfamily of genes, among which at least four families can be discriminated [31]. TSs are only included in one of these families, which encodes for a number of enzymatically active and inactive members [32]. These two forms of TS can be distinguished by the single Tyr_342_His mutation [33]: only the active TSs have the Tyr_342_ residue acting as the enzyme's nucleophile during the ping-pong reaction [34]. TS-mAb competition assays performed with the inactive TS showed that both proteins reacted similarly with the mAb. An equimolar mixture of inactive and active TSs, displayed ∼50% reduction of the neutralizing reactivity (Figure 1D). In a separate set of assays, heat-inactivated TS was not recognized by the mAb 13G9 (Figure S1), consistent with the hypothesis that the neutralizing epitope is conformational [35]. In the infective trypomastigote stages, all TSs include the SAPA *C-*terminal extension [31], which is absent in all the other TS-related families allowing for clear-cut discrimination. To address whether the mAb 13G9 was specific only for TS proteins, extracts from biotinylated trypomastigotes were reacted with the antibody (Figure 1E). Pulled-down material was subjected to Western blot and developed in parallel with anti-SAPA (for TS) and streptavidin for all the biotinylated parasite surface components. Strong signals were readily observed in both lanes, matching the TS expected protein sizes. No differential pattern was detected whatsoever, confirming the very high specificity of 13G9 antibody only towards proteins belonging to the TS family.

### mAb 13G9 Reduces Cell Invasion and Inhibits the Sialylation of the Parasite

**Figure 2:**
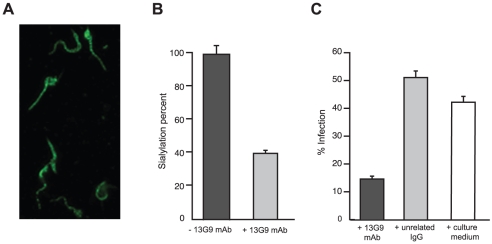
Reactivity of mAb 13G9 with *T. cruzi* parasites. **A**) *T. cruzi* surface labeling by the 13G9 mAb. Epifluorescence microscopy of *T. cruzi* trypomastigotes, seeded onto poly-L-lysine-treated coverglasses, and immunolabeled with 13G9 mAb followed by a secondary FITC-labeled antibody. **B**) Inhibition of parasite sialylation. Trypomastigotes obtained from cell cultures made in ‘low sialyl-donors’ conditions, were sialylated with TS and sialyllactose, in the presence of mAb 13G9. Total sialic acid was quantified by the thiobarbituric acid method and referred to re-sialylated parasites in the absence of mAb as 100% (approximately 1.2 pmoles of sialic acid/10^6^ parasites). **C**) Effect of mAb 13G9 in infection assays on mammalian cells. Parasites were preincubated for 1 h with 13G9 antibody (100 µg/ml) before infection. After 24 hrs, infected cultures were fixed and stained with Hoescht 33342. At least 300 cells were counted.

The reactivity of mAb 13G9 with whole parasites was assayed by immunofluorescence showing surface labeling consistent with the expected cellular membrane localization of TS (Figure 2A). The ability of the mAb to inhibit TS-mediated transfer of sialic acid from the surrounding environment to the parasite's surface molecules was then tested. To reduce the basal sialylation of parasites, sialyl residue donors were largely depleted replacing fetal bovine serum (FBS) by bovine serum albumin (BSA) in the infected tissue cultures; only host cells remained as the unique source of the sugar. Trypomastigotes were then collected and incubated with α(2,3)sialyllactose as sialic acid donor and TS, in the presence of mAb 13G9. The amount of transferred sialic acid was determined by the thiobarbituric acid method [36]. As shown in Figure 2B, mAb 13G9 very efficiently inhibited the parasites' sialylation, demonstrating its biologic relevance as a TS-inhibitory molecule. The sialylation observed in the treated parasites corresponds to the sugar acquired before the addition of the mAb. These quantitative results are in agreement with the Western blot assays we have recently reported for sialyl-transfer inhibition by mAb 13G9 using azido-modified sialic acids [37].

TS is involved in cell invasion [8], [12] given that sialic acid is required for competent interplay with the host cells. The ability of mAb 13G9 to interfere with the invasion process was therefore studied. The addition of the mAb (Figure 2C) strongly reduced the number of infected cells, highlighting its biologic activity and contributing direct evidence that TS is a valid target for drug discovery.

### 3D Structure of the Immunocomplex Fab/TS

To gain atomic insight into the antigen-antibody interactions allowing mAb 13G9 to neutralize the TS catalytic activity with extremely high efficiency, we solved the structure of the immunocomplex by X ray crystallography.

Crystallogenesis screenings were performed under a sitting-drop vapor diffusion setup with a Honeybee963 robotic station, using standard 96-well plates. Several initial hits were obtained. Further manual optimization eventually allowed to grow crystals (0.7×0.05×0.05 mm) in polyethylene glycol (PEG) 20,000 plus dioxane, suitable for X ray diffraction data to be collected (Table 1). Limiting resolution was 3.4Å on a Cu rotating anode generator, and indexing was straightforward, indicating a primitive cell in the trigonal/hexagonal system. Cell parameters (a = b = 178.1Å, c = 140.7Å) suggested the presence of as many as 3 binary complexes per asymmetric unit, raising as well the hypothesis that its weak diffraction could respond to limiting X ray beam intensity in the context of a fairly large unit cell (low number of scattering cells per crystal unit volume). To rule out this possibility, several crystals were tested at the ALS (Advanced Light Source, Lawrence Berkeley National Laboratory, Berkeley, CA) beamline 5.0.2 (8×10^11^ photons/s with 1.5 mrad divergence at 12.4 keV), with no detectable improvement in resolution as judged by standard quantitative statistics, strongly suggesting that crystal disorder linked to high solvent content (66% as determined after full refinement) is the major cause for maximum resolution sphere limitation.

**Table 1:** Data processing and refinement statistics.

	TS-mAb 13G9 complex
Space group	P 3_1_
Wavelength (Å)	1.5418
Data Resolution (Å) a	40–3.4 (3.58–3.4)
Measured reflect.	68611
Multiplicity a	3.6 (3.5)
Completeness (%) a	99.8 (100)
R_meas_ (%)a,b	19 (51.6)
<I/σ(I)>^a^	6.7 (1.5)
a b c (Å)	178.1 178.1 140.7
Refinement resolution (Å)	29.8–3.4
R_cryst_ c [N° refs]	0.165 [67573]
R_free_ c [N° refs]	0.205 [1014]
Rms bonds (Å)	0.013
Rms angles (°)	1.53
Protein non-hydrogen atoms	24414
Water atoms	42
Ligand atoms	13 (2 dioxanes + 1 Na)
Residues in Ramachandran plot regions d(preferred + allowed/outliers)	3109/35
PDB ID	3OPZ

a Values in parentheses apply to the high-resolution shell.

b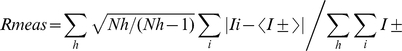; N*_h_*, multiplicity for each reflection; I*_i_*, the intensity of the *i* ^th^ observation of reflection *h*; <I>, the mean of the intensity of all observations of reflection *h*, with 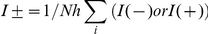; 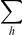 is taken over all reflections; 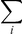 is taken over all observations of each reflection.

c ; 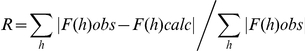 ; R_cryst_ and R_free_ were calculated using the working and test hkl reflection sets, respectively.

d Total refined protein residues equal 3172, from which 28 terminal amino acids (the *N-* and *C-*termini on the 9 chains; plus residues: TS#399, TS#409 (in chains A, B & C), Fab#27, Fab#29 (in chain H), Fab#137, Fab#139 (in chain I), all flanking unmodeled gaps) were not included in the Ramachandran analysis (as implemented in Coot v 0.6.2-pre-1).

No 6-fold peaks were found in self-rotation function maps, and the κ = 180° section revealed significantly weaker signals than the 3-fold axis (data not shown) consistent with point group 3. Systematic extinctions were observed in the reciprocal 00l axis, strongly suggesting space groups P3_1_ or P3_2_. The structure was solved by molecular replacement confirming SG P3_1_. Two search probes were used to calculate rotation and translation functions: Protein Data Base (PDB) 3CLF (mouse IgG Fab fragment, chosen according to sequence similarity to mAb 13G9) and 2AH2 (high resolution *T. cruzi* TS model). Iterative cycles of maximum likelihood refinement [38] were interspersed with manual rebuilding [39]. The high resolution of the molecular replacement search models resulted in excellent maps and straightforward rebuilding, mostly adding missing side chains on the immunoglobulin heavy and light chains. Tight non-crystallographic symmetry restraints were kept only in the first refinement cycles, thereafter allowing for automatic local NCS detection, with variable weights according to evolving rms deviations, as implemented in the program Buster/TNT [40]. Model refinement statistics are summarized in Table 1. Interestingly, the PISA server (European Bioinformatics Institute, Hinxton) predicts that the TS-Fab 13G9 complex would not be stable in solution, contradicting our experimental results. This discrepancy reveals the still challenging task of predicting energetic and thermodynamic properties of protein/protein associations, based on the analysis of crystal structures of partners and derived complexes, despite the fact that prediction algorithms are complex and attempt integrating enthalpic and entropic effects, as well as solvent accessible surface burial and geometric complementarity [41].

Indeed, three binary Fab-TS complexes are located in the asymmetric unit, all very similar at the level of precision of our data. Refined models of immunocomplex 2 (IC2, composed by TS chain B, and chains I and M of the Fab molecule) and IC3 (TS chain C, complexed to Fab J and N) were superposed sequentially onto complex IC1 (TS chain A with H *"heavy"* and L *"light"* chains from the Fab molecule) minimizing root mean squared deviations (rmsd) of atomic coordinates. Such structural alignments resulted in 0.84Å rmsd between IC1 and IC2, and 0.82Å between IC1 and IC3. Regions of highest variation correspond to intrinsically mobile segments, as reflected by detailed analysis of atomic displacement parameters (isotropic B factors). The mean B factor for all atoms is relatively high (59.9 Å^2^), consistent with the low resolution to which these crystals diffract X rays. Crystal packing is indeed loose, leading to high bulk solvent content and corresponding protein flexibility. TS molecules display lower B factors then the Fab dimers to which they are bound. A global tendency is also maintained among the independent complexes, IC3 showing greater mobility than IC2, which in turn is more flexible than complex IC1 (59>53>48 Å^2^), probably due to the different packing environments. In the case of the immunoglobulin heterodimers, chains also display a clear difference among variable domains, more rigid, compared to the constant domains, which show a reproducible flexibility on the distal half, away from the interdomain hinge.

**Figure 3:**
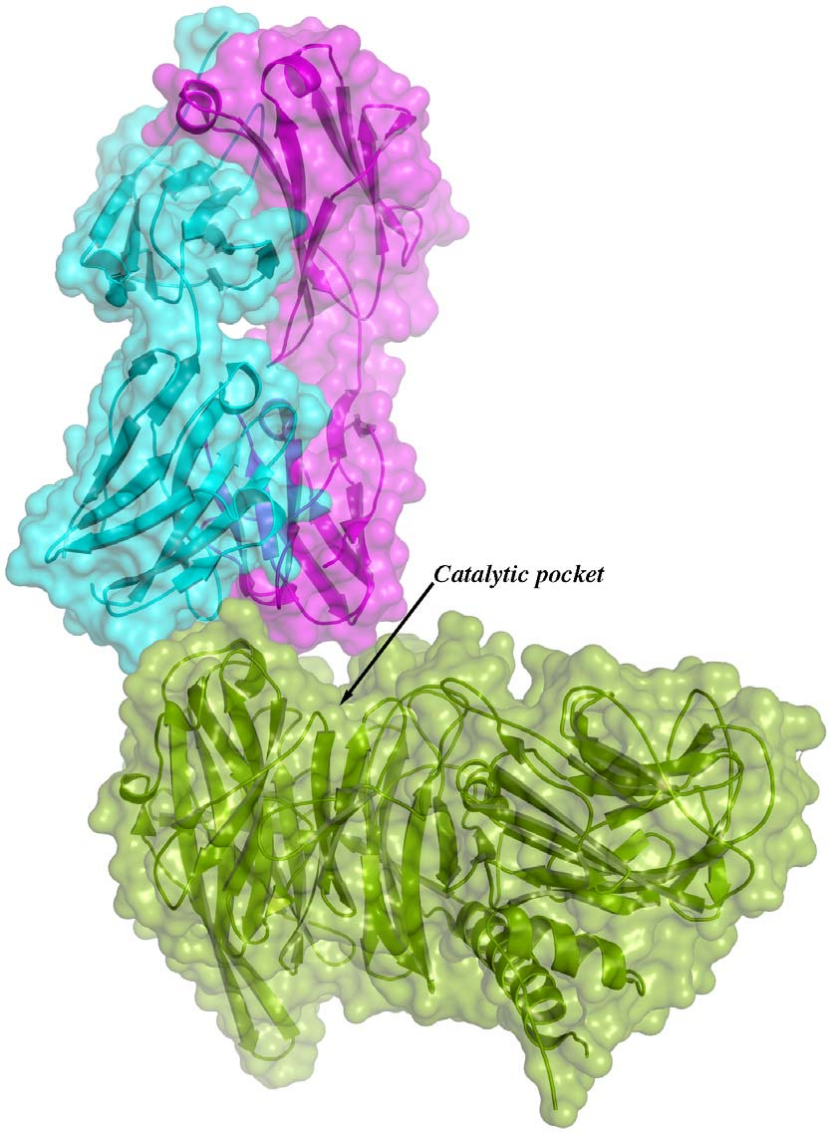
Overall structure of one binary immunocomplex. Immunocomplex 1 (IC1 as defined in the text) is depicted as a background cartoon representation with a superposed transparent solvent-accessible surface rendered in colors. *trans*-Sialidase is colored green, Fab light chain magenta and heavy chain cyan. The antibody light chain is slightly occluding the entrance of the enzyme's catalytic pocket, while the heavy chain is more eccentric, establishing a large interaction surface on one side of the reaction center.

Given the overall structural similarity among the three complexes and the fact that complex IC1 resulted in a model with lower B factors, subsequent analyses will be referred only to this complex. Figure 3 shows the immunocomplex IC1 highlighting that the variable regions of the Fab light chain are interacting with TS loops located closer to the entrance of the enzyme's catalytic pocket, while the heavy chain associates to an adjacent, more distal patch.

**Figure 4:**
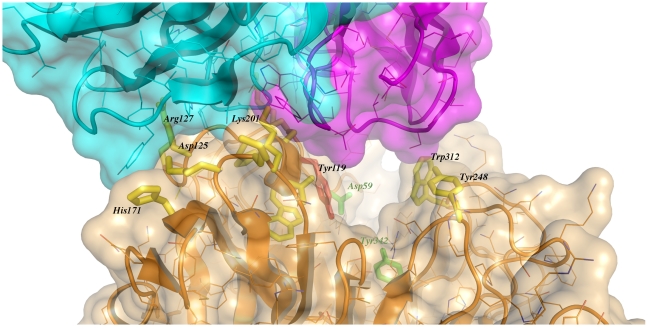
Close-up of the TS/antibody interface. Highlight of the spatial distribution of the epitope residues (in stick representation, colored yellow). TS is shown in orange, light Fab chain in magenta and heavy chain in cyan. For clarity not all the epitope residues are shown nor labeled (see text for full analysis). On top of the cartoon secondary structure representation, residues are represented in lines for the three chains. As a reference for TS positions within the reaction center, the catalytic amino acids Y_342_ (on the floor of the pocket) and D_59_ are highlighted as green sticks; Y_119_ (colored red) forms part of the epitope, normally flexible in free TS. Note how the mAb light chain precludes free mobility of Y_119_, which plays a key function in *trans*-glycosylation.

The solvent accessible surface that becomes buried due to the enzyme-antibody interaction corresponds to 1810.2 Å^2^ (916.5 Å^2^ on the TS and 893.7 Å^2^ on the Fab, adding 506 Å^2^ from the heavy chain, and 387.7 Å^2^ from the light chain), within the typical range of antibodies reacting with protein antigens. On this interface, 15 hydrogen bonds and one salt bridge can be distinguished, as well as a number of residues that establish contact interactions (van der Waals forces), as listed on Table 2. The resolution limit of the diffraction data allowed for the identification of very few water molecules, none of which are directly involved in the accessible nor the buried surfaces engaged in interaction. The shape complementarity statistics [42] correspond to 0.673 and 0.645, after analysis of the interface areas with the light and the heavy chains, respectively. These figures are within the typical range (0.64–0.74) of specific protein:protein interfaces. The epitope (Figure 4) consists of residues H_171_, Y_248_, R_311_–W_312_, and loops 199–201 (KKK) and 116–128 (SRSYWTSHGDARD - W_120_ and A_126_ do not interact directly).

**Table 2:** Residues that display a significant change in solvent-accessible surface, comparing the separate and complexed structures of TS and mAb 13G9.

Heavy chain	Light chain	TS (interface with heavy chain)	TS (interface with light chain)
Tyr 31*	Ser 28	Ser 115 §	Asn 60 §
Asp 32†	Ser 30	Ser 116	Val 91 §
Trp 33*	His 31	Arg 117*	Arg 117*
Tyr 52*	Tyr 48*	Ser 118*	Ser 118
Tyr 57	Ile 49	Ser 122*	Tyr 119
Ile 58	Tyr 52	His 123	Thr 121
Asn 59*	Ser 66*	Gly 124*	Ser 122
Tyr 60	Gly 67	Asp 125*	Lys 200
Arg 98 §	Trp 90*	Arg 127†	Lys 201*
His 100 §	Ser 91	Asp 128	Gln 202
Tyr 101*	Thr 92	His 171	Tyr 248
Asp 102*	Phe 93	Lys 199*	Arg 311*
Gly 103*		Lys 200*	Trp 312
Ser 104*		Lys 201	
Tyr 105 §			

*also establishes hydrogen bonds.

**†:** also establishes salt bridge.

**§:** no direct contact is observed.

**Figure 5:**
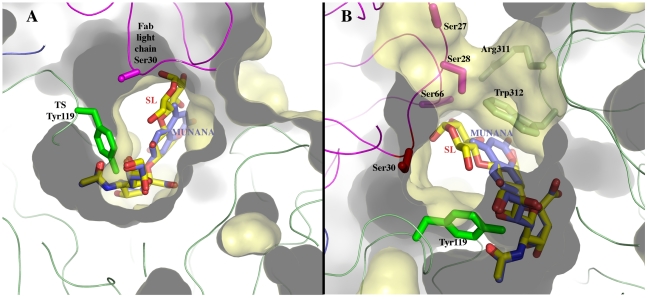
Sialoconjugate substrates modeled in the TS reaction center, in the context of the immunocomplex structure. (**A**) α(2,3)sialyllactose (SL, carbons in yellow) and MU-NANA (carbons in purple) are shown in stick representation, colored according to atom elements (oxygen in red, nitrogen in blue). The carbohydrates are grafted from PDB models 1S0I (for SL) and 1S0J (MU-NANA), after structural superposition of the TS molecules onto the immunocomplex, resulting in their specific positions within the TS catalytic pocket. TS and Fab molecules are shown in ribbons (TS in green, Fab light chain in magenta and heavy chain in blue), with their corresponding solvent-accessible surfaces on top. The surface has been cut to highlight the inner architecture of the TS catalytic pocket: this orientation does not allow appreciating that the site is open from above and beneath the plane of the paper. TS Y_119_ (green sticks) is seen directly obstructing the sialic acid position, and its normal mobility is hindered by antibody's light chain S_30_ (magenta sticks). (**B**) A similar representation as in panel (A), in a rotated orientation scene, to highlight the ‘roof’ formed by residues S_66_–G_67_ of the Fab light chain (in magenta sticks to the top left of the panel) in direct contact with TS residues R_311_–W_312_ (in pale green sticks, to the right of the figure). Note the expected clash of the glucosyl residue in sialyllactose against loop 66–67, and the better fit of the smaller MU-NANA substrate, still quite restricted in free torsional movements. Y_119_ is again shown (strong green sticks), precluding entrance of the sialic acid moiety of both modeled sugar compounds.

The structural bases of the catalytic inhibitory effect that this mAb elicits, can start to be elucidated by modeling the entrance of the sialylated substrate into the TS reactional center in the context of the TS-Fab complex (Figure 5). Superimposing TS PDB models 1S0I and 1S0J, onto our structure, allowed to define the positions of the substrates *N-*acetyl-neuraminyl-lactose (α(2,3)sialyllactose) and 4-methylumbelliferyl-*N*-acetyl-neuraminic acid (MU-NANA), respectively (Figure 5). The most readily observable feature is the steric hindrance that TS residue Y_119_ imposes, blocking the entrance of the sialyl residue in the reactional pocket.

**Figure 6:**
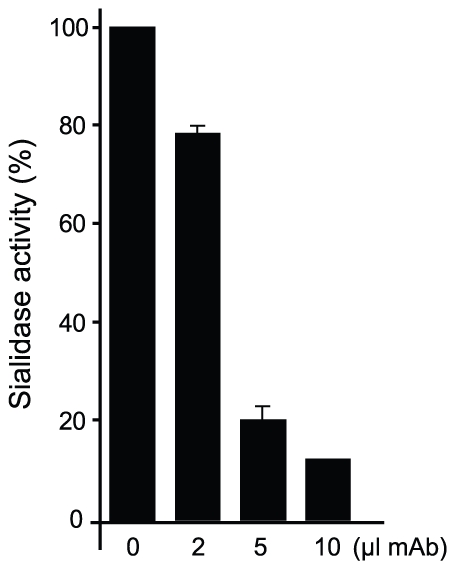
mAb 13G9 inhibits TS-catalyzed MU-NANA hydrolysis. TS (50 ng) was incubated with increasing amounts of 13G9 hybridoma supernatant for 10 min as indicated, then 200 µM MU-NANA was added and incubation continued for 30 min. Controls with complete medium were run in parallel.

The free mobility of the phenolic side chain of Y_119_ is limited by the juxtaposed residue S_30_ from the Fab's light chain (Figure 5). This restraint seems to play a central role in precluding the entrance of sialylated substrates into the catalytic pocket, entrance that absolutely requires the movement of Y_119_ [23]. A second effect could not be excluded, namely the spatial constraint exerted by the overall architecture of the associated complex. Residues S_26_–S_28_ (within the light chain complementarily determining region CDRL1) and S_66_–G_67_ on the same Fab chain, establish direct contact with TS residues R_311_ and W_312_. This interaction is located just on top of the catalytic pocket entrance, functioning as a ‘roof’ (SG/RW roof), where the catalytic center itself would be the floor. As shown in Figure 5B, when sialyllactose is located in position, the substrate pocket appears to be too small, predicting direct clashes of the glucosyl residue with the SG/RW roof (particularly residues Ser_66_–Gly_67_ of the Fab light chain). This scenario of course implies that Y_119_ could eventually be forced to move out of the sialic acid binding site, an unlikely event. The light chain loop 29–31 is also prone to interfere with the saccharide, if rearrangements are to be considered during its accommodation (data not shown). In order to obtain further experimental data evaluating the relative effects of Y_119_-mobility hindrance and/or the spatial constraints exerted by the SG/RW roof onto the catalytic pocket cavity volume, MU-NANA was assayed in TS-catalyzed sialidase reactions. MU-NANA is an artificial substrate that allows for TS-catalyzed hydrolytic and trans-glycosidase activities [43], and given its smaller volume, could better accommodate, avoiding steric clashes with the SG/RW roof structure (Figure 5B). TS-mediated MU-NANA hydrolysis was efficiently inhibited by mAb 13G9 (Figure 6), suggesting that the immobilization of Y_119_ does play a central role. The spatial confinement in the pocket, partly due to the SG/RW roof structure, might impose secondary constraints precluding torsional accommodation, even in the case of smaller compounds.

## Discussion

This report describes an extensive biochemical and structural characterization of the mouse mAb 13G9, which is herein demonstrated to act as a powerful inhibitor of the *T. cruzi* TS catalytic activity, displaying high specificity and affinity for the enzyme. *T. cruzi* TS is a virulence factor required for the survival of the parasite in the mammalian host. Several different biologic activities of the enzyme can be discriminated. The parasite uses TS activity to sialylate its own surface molecules, allowing it to evade lysis by serum factors [9], [10]. In this context, it should be noted that the addition of mAb 13G9 inhibited this sialylation process (Figure 1) in agreement with our previous findings with azido-modified sugars [37]. As well, TS is not only directly involved in the parasite/host cell interaction through the generation of a required sialylated epitope [7], [8] but also in escaping from the parasitophorous vacuole to the cytoplasm [12]. In concert with these findings, here we report that mAb 13G9 significantly reduces parasite infection of cell cultures (Figure 1). Passive transfer of neutralizing mAb 13G9 to heavily infected mice, protects them against TS-induced deleterious effects on the immune system and platelets [5], [17]. In this sense, it is well known that antibodies against neuraminidases are also effective in preventing other diseases such as Influenza [44]. These protective effects are very much promising to delineate a therapeutic tool. The high molecular weight of antibodies constitutes a main drawback in their use, due to eventual hindrance for effective diffusion into infected tissues, where high concentrations of locally produced TS are expected to be found. On the other hand, Fab fragments, small recombinant antibody-derived molecules (e.g. scFv), or yet antibody-mimetic engineered molecules [45], can be cleared exceedingly fast from the bloodstream [46], resulting in poor pharmacokinetic figures. PEGylation, and other modifications to improve bioavailability of these smaller protein scaffolds, constitute interesting approaches to be tested using mAb 13G9 as starting lead [47].

As a second interesting avenue to explore for therapeutic derivatives, the high affinity and specificity of this mAb, prompted us to elucidate its neutralizing mechanism, as an attempt to thereafter conceive low molecular weight inhibitors, suitable as chemotherapy leads. Some information can be gathered in this respect from previous studies of the neuraminidase from Influenza virus, a protein orthologous to TS. The overall geometry of the antibody/TS association that we are now reporting, is reminiscent of the one described for a Fab/Influenza-N2 neuraminidase complex (PDB 2AEP; [48]), which shows interaction with enzyme's loops on the same side of the reaction pocket, opposite to the patch where most other anti-neuraminidase antibodies have been reported (such as the ones involving avian N9 neuraminidase with antibodies NC41 and NC10, PDBs 1NCA and 1NMB, respectively; among others) [49]–[51]. The interaction surfaces of TS-13G9 mAb (this report) and N2NA-Mem5Fab (2AEP) are largely overlapping, although the antibodies are bound in inverted configurations with respect to the location of the heavy and light chains. Well defined escape mutations in Influenza (loops including positions 198–199 and 220–221, following N2 Influenza numbering scheme) identify epidemiologically important antigenic sites of neuraminidase, revealing antigenic drift in human viruses seemingly under natural antibody selection of enzyme variants [52]. These loops, connecting β2–β3 within the second blade of the six-bladed β-propeller domain, and β4 of this blade with β1 of the next one, are not structurally conserved between *T. cruzi* and Influenza enzymes, being longer in the former. Nevertheless, it is clear that the equivalent loops in *T. cruzi* TS do play a critical role in the 13G9 Fab association that we are now reporting.

One of the specific mAb loops that interact in a proximal position to the catalytic pocket of the enzyme, was observed precluding the displacement of Y_119_, a critical residue that has already been shown to be flexible in TS [24], [53]. Indeed, the mobility of Y_119_ plays a key role in the *trans*-glycosidase mechanism of TS. The determination of the three-dimensional coordinates of the paratope, including these features that lead to spatial constraints, uncovers relevant information. This is to be used as a precise guide, not only to undertake peptidomimetic syntheses, but most importantly, to use as a working template for the synthesis of non-peptidic molecules including critical pharmacophores [54].

## Materials and Methods

### Ethics Statement

The protocol of this study was approved by the Committee on the Ethics of Animal Experiments of the Universidad Nacional de San Martín, which also approved protocol development under the recommendations in the Guide for the Care and Use of Laboratory Animals of the National Institutes of Health.

### Recombinant Enzymes

Recombinant *T. cruzi* TSs (constructs 1N1, 2Vo, Δ1443TS and 3.2) [24], [28], [33], *T. rangeli* sialidase [23] and *T. brucei* TS [55] were used. The 1N1 and 2Vo clones correspond to the full-length (including the SAPA repeats [33]) wild type genes that encode for enzymatically active and inactive molecules, respectively. The Δ1443TS recombinant TS was used for immunization procedures. Δ1443TS is an engineered variant where the deletion of a non-neutralizing epitope in the globular domain was done [28]. The TS 3.2 construct [24] is engineered to express the enzymatically competent globular domain only, containing seven mutations of surface-located residues that allow for protein crystallization. All TSs were expressed in *Escherichia coli* BL21 and immediately used after purification, avoiding >3 weeks storage at 4°C. Recombinant proteins were purified to homogeneity as described elsewhere [56], briefly, TS was subjected to immobilized metal affinity chromatography (Ni^++^-charged, Hi-Trap Chelating HP) followed by MonoQ anionic exchange chromatography (both from GE-Healthcare).

### Mice, Immunization Procedures and Neutralizing Titer Determination

C3H/HeJ male animals (60 day old) were used. Mice received three intramuscular doses of Δ1443TS recombinant enzyme [28], 10 µg each with 100 µg of thiophosphodiester backbone CpG-ODN 1826 oligonucleotide (5′-TCCATGACGTTCCTGACGTT-3′, CpG motifs underlined) (Sigma-Genosys) as adjuvant [57]. TS-inhibition assay was performed as previously described [30], preincubating sera with TS and then testing for remnant activity using α(2,3)sialyllactose (Sigma) and [*D-glucose*-1-^14^C]-lactose (GE-Healthcare) as donor and acceptor substrates, respectively. Best responders were selected for cell fusion procedures.

### Hybridoma Screening and mAb Production

Splenocyte suspensions were mixed with Sp2/0-Ag14 cells (ATCC) and fusions performed with polyethylene glycol (GIBCO) following standard procedures [58]. Cells were seeded on 96-well flat-bottom plates at a density of 1×10^5^ cells/well in RPMI 1640 with 2 mM Na Piruvate, 10% FBS, 1X hypoxanthine-aminopterin-thymidine (HAT) solution (all from Invitrogen) and supplemented with 2% supernatant of Sp2/0–Ag14 cultures. One-week later, plates were observed under microscopy and the supernatant of those wells containing hybridomas were taken and refilled with fresh medium. ELISA was performed with these samples in search for TS-specific antibody production. To preserve discontinuos epitopes, the recombinant TS 1N1 containing the *C*-terminus repetitive extension (SAPA) was linked to the plate (MaxiSorb, NUNC) by Protein A-Sepharose (HiTrap, GE-Healthcare)-purified rabbit IgG anti-SAPA, a procedure that safely retained the enzymatic activity (not shown). Those culture wells where anti-TS antibodies were detected were further assayed by TS-inhibition assay [30]. Hybridomas secreting neutralizing antibodies were cloned twice by cell dilution. From four inhibitory antibody-secreting hybridomas detected, only one (named 13G9) was successfully recloned twice by the dilution method and then expanded. The mAb 13G9 was typed as IgG2aκ using the Mouse Antibody Isotyping Kit (GIBCO).

### mAb Production and Purification

The 13G9 hybridoma was cultured in RPMI 1640 plus 2 mM Na Piruvate and 10% FBS. Supernatants were clarified and subjected to Protein A-Sepharose (GE-Healthcare) affinity chromatography. The mAb was eluted with 150 mM NaCl, 0.1 M Glycine-HCl pH 3.5 and aliquots were received on 0.1 M Tris-ClH pH 7.6 and dialyzed against 50 mM NaCl, 20 mM Tris-HCl, pH 7.6. Fractions were then loaded into an ion-exchange column (MonoQ, GE-Healthcare) and eluted with a 50–500 mM NaCl gradient in the same buffer (Figure S1). Purified 13G9 mAb was tested by TS-inhibition assay [30] and by reactivity to native and denatured TS-SAPA molecules spotted on nitrocellulose (Figure S1).

### Sequence Analysis

cDNA was obtained from 13G9 hybridoma cultures from total RNA using the SuperScript II retrotranscriptase (Invitrogen). cDNA quality control was performed by GAPDH amplification. To amplify the immunoglobulin Fab chains, oligonucleotide primer sets Fwh1 (5′-GTCAGGAGTTGAGCTGGTAAG-3′), Fwh2 (5′-CCTGGGACTTCAGTGAAGATG-3′) and Rvh (5′-TGGAGGACAGGGCTTGATTG-3′) were used for the heavy chain, and Fwl1 (5′-AACAATCATGTGTGCATCTATA-3′), Fwl2 (5′-GAGGAGATCACCCTAACCTG-3′) and Rvl (5′-TCAGGATGTGGTTGCAACAC-3′), for the light chain. *Pfu* DNA polymerase (Promega) was used and amplicons cloned and sequenced.

### Determination of Kinetic Parameters of mAb 13G19 Reactivity

The association/dissociation kinetic constants (*k*_on_/*k*_off_) were determined with a BIAcore 2000 (BIAcore AB, Uppsala, Sweden). Purified mAb was dialyzed against 20 mM sodium acetate pH 5.6 and immobilized to sensor chips CM5 by using the amine-coupling kit (BIAcore AB). Chips were quenched with 1 M ethanolamine/HCl. After equilibration with 150 mM NaCl, 0.05% P20 surfactant, 10 mM HEPES pH 7.4 (HBS-EP), different concentrations of TS (from 1 nM to 10 µM) were injected at 50 µl/min. After each recording cycle, chips were regenerated with an injection of 2 mM HCl for 30 sec. A free surface of the chip was used as control throughout the experiments. Kinetic constants were evaluated using the program BIAevaluation 3.01 (BIAcore AB). Isothermal titration calorimetry assays were performed in the laboratory of Dr. Alan Cooper (Department of Chemistry, Joseph Black Building University of Glasgow, UK).

Inhibition constants of TS activity were determined for mAb 13G9 and its derived Fab fragment (see below for digestion details) by testing increasing amounts of inhibiting antibody with 2 ng of TS in 30 µl of 150 mM NaCl, Tris-HCl pH 7.6. After 5 min at room temperature (RT), 1 mM sialyllactose and 0.4 nmol (about 40,000 cpm) of [*D-glucose*-1-^14^C]-lactose (54.3 mCi/mmol, GE-Healthcare) were added. Remnant TS activity was evaluated [30] after 30 min incubation at RT.

### Specificity of mAb 13G9 Reactivity

Trypomastigotes (120×10^6^) were purified from supernatants of infected Vero cell cultures, biotinylated (Sulfo-NHS-LC-Biotin kit form Pierce, Rockford, IL) washed and lysed in the presence of protease inhibitors and centrifuged at 16,000 g. Supernatant was precleared with Protein A-Sepharose (GE-Healthcare) and then reacted with 50 µl of mAb 13G9 hybridoma supernatant for 30 min. Then, Protein A-Sepharose was added and beads extensively washed before SDS-PAGE sample buffer addition and boiling. SDS-PAGE was performed with two parallel aliquots that were then transferred to polyvinylidene fluoride (PVDF) membrane (GE-Healthcare) and developed with either rabbit IgG anti-SAPA followed by horseradish peroxidase (HRP)-labeled secondary antibody or HRP-streptavidin and Super Signal West Pico Chemiluminescent substrate (Pierce).

### Inhibition of Parasite Cell Invasion

*T. cruzi* trypomastigotes (CL-Brenner strain) obtained from Vero cell cultures (Minimum Essential Medium (Invitrogen) supplemented with 0.2% BSA instead of FBS to reduce sialic acid donors) were exhaustively washed with PBS. Parasites were tested by infection of Vero and HeLa cell cultures in the same medium at a multiplicity of infection of 30 in the presence of 0.1 mg/ml of mAb 13G9. After 3 h, cells were washed and medium plus 10% FBS was added. Cells were fixed and stained 24 h later for counting infected cells under microscopy. IgG purified from naïve mouse was used as control.

### Inhibition of Parasite Sialylation

Parasites obtained under low sialic acid conditions as above were incubated with 1 mM sialyllactose (Sigma) as sialyl residue donor substrate and TS (2 µg/ml) with or without mAb 13G9 (0.1 mg/ml). After washings with PBS, sialyl residue content was determined by the thiobarbituric HPLC assay after hydrolysis in 0.1 M HCl for 1 h at 80°C [36]. IgG purified from naïve mouse was used as control.

### Immunofluorescence

Cell culture-derived trypomastigotes were washed with PBS and incubated with mAb 13G9 (0.05 mg/ml) for 15 min, washed, fixed with 1% paraformaldehyde for 10 min on ice, washed again and blocked for 1 h with 2% BSA plus 5% swine serum in PBS. After that, the parasites were adhered to glass slides *via* Poly-*L*-Lysine (Sigma), blocked again, developed with a FITC-conjugated secondary antibody (DAKO, Denmark) and observed by epifluorescence microscopy.

### Inhibition of Sialidase Activity

The sialidase activity of TS was determined by measuring the fluorescence of 4-methylumbelliferone released by the hydrolysis of 0.2 mM MU-NANA (Sigma). To 50 ng of TS, different amounts of hybridoma culture supernatant (0–10 µl) or RPMI plus 10% FBS (control) were added. The assay was performed in 50 µl of 150 mM NaCl, 20 mM Tris-ClH pH 6.8. After 10 min at RT, 200 µM of MU-NANA was added and incubation continued for 30 min. The reaction was stopped by dilution in 0.2 M NaHCO_3_ pH 10, and fluorescence was measured with a DYNA Quant TM 200 fluorometer (GE-Healthcare). Fluorescence values were referred to each RPMI control.

### Generation of Antibody Fragments and Immunocomplex

Purified mAb was dialyzed against 2 mM EDTA, 0.1 M Tris-HCl pH 7.6. Before papain digestion 1 mM dithiothreitol (DTT) was added. Papain-agarose beads (Sigma) were washed with the same buffer and activated by addition of 1 mM DTT for 15 min at 37°C. The Fab fragment was generated by digestion for 5 h at 37°C with papain-agarose beads (3U papain/mg mAb; 30 mg of beads for 14 mg of mAb) with gentle end-over-end agitation [58]. After centrifugation at 3,000 rpm, 10 µM *trans*-epoxysuccinyl-*L*-leucylamido(4-guanidino)butane (E-64) was added. Undigested antibody and Fc fragment were depleted by Protein A-Sepharose (GE-Healthcare) chromatography and Fab digestion and purity was assayed by SDS-PAGE.

To generate the immunocomplex, pure TS (3.2 clone) was immediately added after the depletion of papain-beads and E-64 addition step before subjecting the mixture to Protein A-Sepharose chromatography as above (Figure S1). The immunocomplex was brought to 25 mM NaCl and concentrated on a BIOMAX 30 K (Millipore) to 14 mg/ml and the buffer changed to 25 mM NaCl, 20 mM Tris-HCl pH 7.6. The purified immunocomplex was essentially free from contaminating proteins and only traces of TS activity remained (see Figure S1). Before crystallization trials, the immunocomplex was repurified by size exclusion chromatography (Superdex200 10/300, GE Healthcare) in an AKTA Purifier, (GE Healthcare) with isocractic elution in 100 mM NaCl, 20 mM Tris-HCl pH 7.6. The resulting single symmetric peak was pooled and concentrated to 7.5 mg/ml by ultrafiltration (Vivaspin, Sartorius-Stedim Biotech; 30 kDa-cutoff membrane) in buffer 25 mM NaCl, 20 mM Tris-HCl pH 7.6.

### Immunocomplex Crystallization

Crystallogenesis conditions were screened with a HoneyBee 963 robot (Digilab), using the vapor diffusion method in sitting-drops and reservoirs filled with 150 µl mother liquors (kits JCSG Core Suites I, II, III and IV, Qiagen), rendering 396 different conditions in 96-well plates (3-drop round bottom, Greiner). Protein drops were dispensed mixing equal parts of protein and reservoir solutions (300 nl + 300 nl). Plates were immediately sealed and incubated at 20°C. Hits were obtained in several conditions, one of them was chosen for manual optimization in 24-well plates (VDX, Hampton Research). Final optimized conditions consisted in 2+2 µl hanging-drops, 0.1 M bicine pH 8.5, 10% PEG 20,000, 4% 1,4-dioxane as mother liquor. To obtain larger crystals suitable for single crystal X ray diffraction experiments, repeated macroseeding cycles proved to be essential. Each cycle included selection of best crystal seeds that were transferred to protein-free drops of mother liquor and crystals etched for 30 sec (this washing procedure was repeated three times). Finally, the seed was added to a fresh hanging-drop containing 2 µl protein + 2 µl mother liquor, over 1 ml pure mother liquor. Single needles grew in 5–10 days, cryoprotected with mother liquor containing 12% PEG 20,000 and 30% glycerol and flash frozen in liquid nitrogen until data collection.

### Crystal Structure Determination

Single crystal X ray diffraction experiments were performed with a rotating copper anode (Micromax007-HF, Rigaku), multilayer mirrors (Varimax HF, Rigaku) and an image plate detector (Mar345 dtb, Mar Research). Crystals were mounted to collect data under cryogenic temperature (108°K, Cryostream Series 700, Oxford Cryosystems). To attempt improving diffraction resolution, similar crystals were subjected to X ray diffraction using synchrotron radiation at beamline 5.0.2 ALS, equipped with a wiggler inserted device. All data sets were processed with MOSFLM [59], SCALA and TRUNCATE [60].

The structure was solved by molecular replacement with the program Phaser [61], using the models 3CLF (mouse IgG Fab) and 2AH2 (*T. cruzi* TS in complex with 3-flourosialic acid) as search probes. The Fab probe was previously modified using Chainsaw [60], keeping only the conserved side chains, the rest pruned to alanine or glycine.

The model was refined to the highest collected resolution (3.4 Å) with the program Buster/TNT [38], using a maximum likelihood target function and non-crystallographic restraints throughout the entire process. A TLS model was used to refine correlated anisotropic atomic displacement parameters in large rigid-body domains. Reciprocal space refinement cycles were iterated with manual model rebuilding [39]. Validation tools within Coot were inspected regularly during the refinement process. Last validation steps were done with MolProbity [62].

### Accession Numbers

The atomic coordinates and structure factors of the Fab-TS immunocomplex that we have solved in this report are accessible in the PDB with accession code 3OPZ. The models used to solve the phase problem have PDB accession codes 3CLF (mouse IgG Fab fragment) and 2AH2 (*T. cruzi* TS). A certain number of sialidase and *trans*-sialidase structures solved previously by us or by other groups, are mentioned in the Discussion section and can be accessed in the PDB with codes: 2AEP (Fab/Influenza-N2 neuraminidase complex); 1NCA (avian N9 neuraminidase complexed with antibody NC41); 1NMB (avian N9 neuraminidase complexed with antibody NC10); 2AEP (N2NA-Mem5Fab); 1S0I (*T. cruzi* TS in complex with sialyllactose) and 1S0J (*T. cruzi* TS in complex with MUNANA). Sequence of *T. cruzi trans*-sialidase can be accessed from the GenBank with the code L26499.

## Supporting Information

Figure S1 **Production of the mAb 13G9-TS immunocomplex. A**) MonoQ-chromatogram of Protein A-purified hybridoma 13G9 supernatant. The mAb eluted as a single peak. **B**) TS reactivity of eluted and pass-trough proteins. Nitrocellulose membranes were spotted with TS-SAPA native (1) or heat-denatured (2). Upper panel was tested with flow through proteins, middle panel with the eluted peak and lower panel with an anti-SAPA mAb. Filters were developed with an HRP-labeled secondary antibody against mouse immunoglobulins. Note the absence of reactivity to the denatured protein by the 13G9 mAb (middle panel, spot 2) in contrast with the anti-SAPA mAb that recognizes a continuous epitope (lower panel). **C**) Purification of the Fab-TS complex through a Protein A affinity column. The retained protein corresponds to the Fc fraction. **D**) SDS-PAGE of the purified TS-Fab complex. **E**) Almost null remnant TS activity was found in the TS-Fab complex.
(EPS)
